# Identification of functional rare variants in genome-wide association studies using stability selection based on random collapsing

**DOI:** 10.1186/1753-6561-5-S9-S56

**Published:** 2011-11-29

**Authors:** Xin Huang, Yixin Fang, Junhui Wang

**Affiliations:** 1 Division of Public Health Sciences, Fred Hutchinson Cancer Research Center, Seattle, WA 98109, USA; 2 Division of Biostatistics, School of Medicine, New York University, New York, NY 10016, USA; 3 Department of Mathematics, Statistics, and Computer Science, University of Illinois at Chicago, Chicago, IL 60607, USA

## Abstract

Genome-wide association studies are a powerful approach used to identify common variants for complex disease. However, the traditional genome-wide association methods may not be optimal when they are applied to rare variants because of the rare variants’ low frequencies and weak signals. To alleviate the difficulty, investigators have proposed many methods that collapse rare variants. In this paper, we propose a novel ranking method, which we call stability selection based on random collapsing, to rank the candidate rare variants. We use the simulated mini-exome data sets of unrelated individuals from Genetic Analysis Workshop 17 for the analysis. The numerical results suggest that the selection based on a random collapsing method is promising for identifying functional rare variants in genome-wide association studies. Further research to examine the error control property of the proposed method is underway.

## Background

Genome-wide association studies (GWAS) are a powerful approach to identifying common variants associated with complex disease under the common disease/common variant hypothesis. This hypothesis assumes that common variants of small to modest effect are responsible for common diseases [1]. However, recent studies have revealed that the common variants explain only a small proportion of the heritability [2]. Some studies suggest that rare variants, typically defined as variants with minor allele frequency (MAF) less than 5%, are more likely to be functional variants [3,4]. This leads to the hypothesis that the complex disease is associated with both common and rare variants. However, rare variants were not the focus in early GWAS because of the cost of the genotyping technology. Recently, next-generation sequencing technologies have provided cost-effective procedures to detect rare variants and have raised the challenge of how to effectively identify functional rare variants in GWAS.

Many studies have shown that standard statistical methods are not appropriate for identifying functional rare variants because of these variants’ low frequencies and weak signals. To alleviate the difficulty, investigators have proposed collapsing methods, which collapse rare variants within a genetic region of interest, and these methods have become popular (e.g., [5-9]). By collapsing multiple rare variants, the association signals within the region of interest can be enriched and then standard association tests can be applied; see Dering et al. [8] for an overview. Here, we briefly describe the combined multivariate and collapsing (CMC) method proposed by Li and Leal [9]. To perform the CMC method, rare variants are first divided into subgroups by some predefined criterion (say, MAF); then variants are collapsed within each subgroup; finally, a multivariate test is applied. However, the choice of the collapsing criterion seems to be subjective, and an inappropriate collapsing criterion may result in low power.

In this paper, instead of predefining a collapsing criterion, we propose a novel ranking method that is based on random collapsing. We call this method stability selection based on random collapsing (SORC). The proposed method is applied to the simulated mini-exome data sets of unrelated individuals with phenotype Q1 from Genetic Analysis Workshop 17 (GAW17). The numerical results demonstrate that this method can recover many true functional rare variants in the simulation models.

## Methods

### Collapsing methods

Suppose that in a genome-wide association study there are *N* individuals and *P* rare variants located in *K* genes. According to Li and Leal [9], the rare variants within gene *k* are divided into *J_k_* subgroups based on some predefined criterion (say, MAF). In each subgroup, the rare variants are collapsed into an indicator variable:(1)

where *k* = 1, …, *K* and *j* = 1, …, *J_k_*. For those genes with only one variant, no collapsing is necessary. Based on these indicator variables, statistical analysis, such as univariate tests, multivariate tests, and linear regression, can be conducted to identify the functional rare variants.

### Random collapsing

To illustrate the idea of random collapsing, let *J_k_* = 2 (*k* = 1, …, *K*) for simplicity. Assume that there are *M_k_* rare variants within gene *k*. First, an integer *S_k_* is randomly drawn from {1, 2, …, *M_k_*}. Second, *S_k_* rare variants are randomly selected from *M_k_* rare variants as the first subgroup, and the rest of the rare variants constitute the second subgroup. Third, the rare variants in each subgroup are then collapsed into an indicator variable by means of a coding system (Eq. (1)). Finally, standard statistical analyses, such as univariate tests, multivariate tests, and linear regression, can be conducted based on these indicator variables. As opposed to the CMC method, which requires a predefined collapsing criterion, the proposed random collapsing process circumvents the difficulty by repeating the random collapsing multiple times, and a ranking method based on stability selection across all replications can be developed.

### SORC method

In statistical literature, original stability selection [10] is a method that combines a random subsampling procedure with some variable selection algorithm, under the rationale that important variables are more likely to be selected across different subsamples. Borrowing the idea of stability selection, in the proposed SORC method we combine the random collapsing procedure with some variable selection algorithm, say, the least absolute shrinkage and selection operator (LASSO) [11]. Note that the SORC method is different from stability selection [10] in that the randomness is imposed on the collapsing criteria instead of subsampling.

When the SORC procedure is performed, for each repetition of the random collapsing, the rare variants in each gene are randomly collapsed into two indicator variables. Then the LASSO is used to select a subset of variants by minimizing:(2)

where *Y* is the vector of phenotypes, *X* is the matrix of collapsed indicator variables, *U* is the matrix of uncollapsed common variants, *β* and *γ* are linear coefficient vectors for X and U, respectively. The regularization parameter *λ* is chosen using the cross-validation procedure, and the variants being selected are recorded. After *R* repetitions of the random collapsing, for each variant, the relative frequency that it survives the LASSO selection is obtained. According to Meinshausen and Buhlmann [10], this relative frequency is called stability. A list of ranked variants can then be obtained by means of the ordered stabilities. We can report those variants with the largest *T* (say, top 10 or 15) stabilities as suspected functional variants, which are suspected to be associated with the phenotype of interest. Therefore the proposed SORC method is essentially a ranking method that ranks the rare variants based on their corresponding selection stability. However, if one is interested in estimating the type I error (or controlling the family-wise error rate), then further research is needed for determining *T*.

## Results and discussion

We analyzed the mini-exome data set of unrelated individuals simulated by GAW17 following the pilot3 study of the 1000 Genomes Project, which consists of 24,487 autosomal SNPs on 3,205 genes [12]. There are 21,355 rare variants, of which 13,572 are nonsynonymous. There are 200 replicates of phenotypes, including one disease trait and three quantitative traits (Q1, Q2, and Q4) simulated from a selection of designated sequence variants, and other covariates such as sex, age, and smoking status. Throughout our analysis, we coded each variant as 0 or 1 according to the absence or presence, respectively, of minor alleles.

We applied the SORC method to Q1. The linear model is assumed to be the basic association model. We entered only the nonsynonymous SNPs as candidate variants in our model and did not include any covariates. We defined the rare variants as the ones with MAF < 5%. For each replicate of the simulated phenotypes, we preformed 100 repetitions of the proposed random collapsing; the LASSO procedure was implemented with the R package glmnet [13]. Table 1 presents the top 10 genes ranked by the stabilities of the variants contained within them, using the phenotype Q1 in the first simulated replicate. The proposed method identified 39 variants in the top 10 genes, among which 16 are true functional variants used in the simulation model.

**Table 1:** Top 10 ranked genes for trait Q1 in replicate 1

Gene	Functional SNPs	MAF	*β*^b^	Stability^c^
*FLT1*^a^	C13S431	0.02	0.74	1
	C13S522	0.03	0.62	1
	C13S523	0.07	0.65	1
	C13S320	<0.01	0.2	0.95
	C13S524	<0.01	0.62	0.94
	C13S399	<0.01	0.4	0.92
	C13S567	<0.01	0.17	0.88
	C13S505	<0.01	0.45	0.87

*BRWD1*	–	–	–	1

*KDR*^a^	C4S1884	0.02	0.3	0.97
	C4S1889	<0.01	0.94	0.72
	C4S1877	<0.01	1.08	0.67
	C4S1890	<0.01	0.42	0.66
	C4S1873	<0.01	0.58	0.64
	C4S1874	<0.01	0.47	0.63
	C4S1879	<0.01	0.62	0.63

*C14ORF159*	–	0.18	–	0.79

*C1ORF122*	–	<0.01	–	0.7

*ZNF502*	–	0.24	–	0.79

*VEGFA*^a^	C6S2981	<0.01	1.2	0.62

*HNRPUL1*	–	<0.01	–	0.58

*FMNL3*	–	<0.01	–	0.56

*AIF1*	–	0.05	–	0.49

^a^ Genes containing at least one true functional variant.

^b^ True effect size in the simulated model.

^c^ Estimated from 100 repetitions of randomized collapsing.

Table 2 shows the top 13 most identified genes for Q1 across all the 200 simulated replicates, ranked by the number of times they were identified (genes ranked in the top 15 are treated as identified). Among the top 13 most identified genes, 4 contain true functional variants. Table 3 indicates the number of successful identifications of true genes that contain at least one true functional variant for Q1 across all 200 replicates. Although two true genes were never detected for all replicates with the proposed method, about half of the true genes were identified in most of the replicates.

**Table 2:** Top 13 most identified^a^ genes for the trait Q1 across all 200 replicates

Rank	Gene	Number of times selected
1	*FLT1*^b^	200
2	*KDR*^b^	137
3	*ARNT*^b^	60
4	*TACC2*	36
5	*RAD54B*	30
6	*ACP1*	28
7	*C9ORF66*	26
7	*JAK1*	26
9	*CES1*	24
9	*HYAL3*	24
9	*OR2T34*	24
9	*LYPD2*	24
9	*VEGFA*^b^	24

^a^ Genes ranked in the top 15 are treated as identified.

^b^ Genes containing at least one true functional variant.

**Table 3:** Number of times the true genes for Q1 across all 200 replicates are identified^a^

Gene	Rank	Number of times identified
*FLT1*	1	200
*KDR*	2	137
*ARNT*	3	60
*VEGFA*	9	24
*VEGFC*	54	9
*ELAVL4*	122	5
*HIF3A*	608	1
*FLT4*	–	0
*HIF1A*	–	0

^a^ Genes ranked in the top 15 are treated as identified.

For comparison, we also applied three existing approaches to the same data set. The first approach is the single-marker test in which a univariate test is performed to test each variant individually. The second approach is the collapsing method, in which rare variants in the same gene are collapsed by means of a coding system (Eq. (1)) and then a univariate test is performed based on the collapsed variables. The third approach is the CMC method, in which rare variants are collapsed in the same gene and common variants are kept the same and then a multivariate test is applied to each gene. These three approaches have been fully studied and compared by Li and Leal [9]. For each approach, the variants are ranked by −log_10_(*P*-value). Table 4 presents the top 10 genes with the highest ranked variants from the three approaches. For all three methods, among the top 10 genes only gene *FLT1* contains true functional variants.

**Table 4:** Comparison of ranking approaches

Rank^a^	Single-marker test	Collapsing method	CMC method	SORC method
1	*FLT1*^b^	*TBX18*	*FLT1*^b^	*FLT1*^b^
2	*OR2T34*	*FLT1*^b^	*TBX18*	*BRWD1*
3	*LRRK2*	*AMPD3*	*AMPD3*	*KDR*^b^
4	*BRCA1*	*C8ORF31*	*C8ORF31*	*C14ORF159*
5	*PPP1R14BP1*	*SLCO1A2*	*ADAM7*	*C1ORF122*
6	*HSZFP36*	*ADAM7*	*TMEM67*	*ZNF502*
7	*C9ORF66*	*C9ORF66*	*SBF2*	*VEGFA*^b^
8	*ABL2*	*AIF1*	*KIAA0802*	*HNRPUL1*
9	*AIF1*	*SBF2*	*AIF1*	*FMNL3*
10	*RUNX2*	*FARP1*	*FARP1*	*AIF1*

^a^ Genes are ordered by the rank of their SNPs’ −log_10_(*p*-value).

^b^ Genes containing at least one true functional variant

## Conclusions

Our proposed method provides a novel approach to ranking suspected functional rare variants in GWAS. The idea is motivated by the stability selection of Meinshausen and Buhlmann [10]. The result is promising, but some questions still remain unanswered, for example, how many variants should be selected as functional using the ranked stabilities and whether or not there is an error control theorem for the family-wise error rate. In addition, the SORC method can be constructed using other variable selection procedures (e.g., [14]) instead of the LASSO, and it can also be constructed using other collapsing procedures (e.g., [8]) instead of random collapsing. Hence further studies should be done to evaluate and compare the performance of these alternatives.

## Competing interests

The authors declare that there are no competing interests.

## Authors’ contributions

XH, YF and JW discussed the idea of SORC. XH and YF performed the statistical analysis and drafted the manuscript. JW helped to revise the draft. All authors read and approved the final manuscript.
